# The Effect of Temperature on Molecular Structure of Medium-Rank Coal via Fourier Transform Infrared Spectroscopy

**DOI:** 10.3390/ma16206746

**Published:** 2023-10-18

**Authors:** Meng Wu, Yong Qin, Yunhu Qin, Naicen Xu, Lele Feng

**Affiliations:** 1 Jiangsu Mineral Resources and Geological Design and Research Institute, China National Administration of Coal Geology, Xuzhou 221006, China; xzqinyunhu@163.com; 2 Key Laboratory of Coalbed Methane Resources and Reservoir Formation Process, Ministry of Education, China University of Mining and Technology, Xuzhou 221008, China; 3 Nanjing Geological Survey Center, China Geological Surve, Nanjing 210016, China; xncsynge@163.com; 4 School of Safety and Engineering, China University of Mining and Technology, Xuzhou 221116, China; fenglele@cumt.edu.cn

**Keywords:** medium-rank coal, FTIR, functional groups, heat-treated, molecular structure

## Abstract

Fourier transform infrared spectroscopy (FTIR) was used to study the molecular structure of four medium- and low-temperature heat-treated medium-rank coals. The FTIR spectral parameters, which consist of CH_2_/CH_3_, aromaticity (*f_a_*), aromatic carbon rate (*f_C_*), aromatic hydrogen rate (*f_H_*), oxygen-containing (C–O) rate (*IR*), organic matter maturity (*M*), and the degree of aromatic condensation (*Dc*), indicate different characteristics, including changes in the aromatic hydrocarbon structure, fatty hydrocarbon structure, hydroxyl structure, and oxygen-containing functional groups of medium-rank coal. The results show that with the increase in heat treatment temperature, the sulfur content in coal gradually decreases, but the C/H ratio gradually increases. Meanwhile, the content of kaolinite and pyrite in coal gradually decreases, whereas the content of dolomite and hematite gradually increases. With the increase in heat treatment temperature, the relative content of ether oxygen hydroxyl groups in the hydroxyl structure significantly decreases, but the relative content of self-associated hydroxyl groups increases. The relative content of alkyl ether (C–O) in oxygen-containing functional groups gradually increases, whereas the relative content of aromatic nucleus C=C vibration presents a trend of first increasing and then decreasing. In addition, –CH_2_– is the majority in the structure of fatty hydrocarbons, and the absorption peak intensity of asymmetric –CH_3_ stretching vibration increases with the increase in heat-treated temperature. The structure of aromatic hydrocarbons mainly consists of four substituted benzene rings (except for R-303.15 K), in which the relative content of the trisubstituted benzene ring decreases with the increase in heat treatment temperature. With the increase in the heat-treated temperature of medium-rank coal, *Dc*, *f_H_*, *f_C_*, and *f_a_* show a trend of first increasing and then decreasing, *M* and *IR* reveal a trend of first decreasing and then increasing, and CH_2_/CH_3_ present a gradually decreasing trend. In conclusion, during the increase in the heat treatment temperature of medium-rank coal, the length of the fatty side chains in the fatty hydrocarbon structure becomes shorter, the number of branch chains continuously increases, and the maturity and condensation degree of organic matter first increases and then decreases. On this basis, further research on the effect of coal gasification suggests combining various technologies such as ^13^C NMR, XRD, and TG-MS to obtain semi-quantitative structural information of molecules in coal from different perspectives.

## 1. Introduction

Coal is an uneven aggregate mainly formed by cross-linked molecular networks of organic components [1], and its molecular structure undergoes rapid changes during heating [2,3,4]. However, research on the molecular structure and functional group characteristics of coal has always been a focus of attention due to its complex microstructure [5,6,7]. Fourier transform infrared spectroscopy (FTIR), as a non-destructive structural characterization technique, is usually suitable for studying the degree of coalification, functional groups, material composition, and genetic types of coal [8,9,10,11,12,13,14,15]. This technology has the advantages of simple operation, low cost, and effective recognition of cross-linked molecular information of organic substances [4,16,17]. Moreover, the study of molecular structure in coal has always been a subject of fierce debate, including the attribution and distribution characteristics of aromatic, aliphatic, hydroxyl, and oxygen-containing functional groups in coal [18,19,20], as well as the impact of pyrolysis temperature on functional groups of different coal types [21,22]. In addition, the molecular structure parameters of low-rank coal are determined by factors such as depositional environments, microbial abundance, coal-forming plants, mineral composition, and coalification [4,20,23,24,25,26,27].

The high-temperature pyrolysis reaction of coal is used to extract tar, CO, CO_2_, and other products, which can effectively expand the comprehensive utilization value of low-grade coal resources [28]. Notably, the pyrolysis characteristics of lignite between 293.15 K and 923.15 K were studied using FTIR. It was found that the aromaticity increased with increasing temperature [29]. Moreover, FTIR analysis on coal with different degrees of coal carbonization was conducted. It was found that with the increase in coal carbonization degree, the aromaticity and aromatic core structure content of coal increased, while the content of fatty structure and C=O type structure (found at 1710 cm^−1^) decreased [10]. In addition, FTIR was used to study the changes in active groups in coal from 303.15 K to 483.15 K. It was found that carbon and oxygen-containing groups in aliphatic groups were affected to varying degrees [30]. Then, FTIR spectroscopy was used to study the changes in aromatic ring structure (900–700 cm^−1^) and oxygen-containing functional groups (1800–1000 cm^−1^) of low-rank coal during slow heat treatment [31]. Moreover, FTIR was used to analyze the molecular structure changes in six types of coal chars at 723.15 K–973.15 K. It was found that the aromaticity of coal chars increased with increasing coalification degree while fatty hydrogen decreased with the increase in temperature [17]. In addition, FTIR analysis was adopted to indicate that the degree of aromatization of coal increased with the increase in coal rank while aliphatic and oxygen-containing functional groups decreased [32]. Lignite was found to have a high content of CH_2_, indicating that there are fewer side chains of fatty hydrocarbons in coal, and most of them appear in the form of long chains [33].

Currently, FTIR research on the molecular structures of different coal ranks is mainly focused on high-temperature pyrolysis [34,35], but the low-temperature pyrolysis of medium-rank coal is rarely researched [32,36,37,38]. Meanwhile, exploring the evolution of pyrolysis molecular structure can be helpful in optimizing the comprehensive utilization direction of coal, such as dry distillation, gasification, and liquefaction [39,40]. Taking the medium-rank coal from Renjiazhuang Coal Mine in Ningxia as the research object, this paper used 303.15 K, 523.15 K, 623.15 K, and 723.15 K heat-treated coal samples. Through FTIR experiments and peak fitting techniques, the relative content of functional groups of different heat-treated coal samples was calculated. In combination with seven infrared spectral molecular structure parameters, the molecular structure characteristics of low-order coal with different heat treatments were comprehensively analyzed.

## 2. Analytical Methods

### 2.1. Coal Samples

The sample was collected from the No. 9 coal seam of the Taiyuan Formation in Renjiazhuang Coal Mine, Eastern Ningxia, China. During the entire process, from collection to transportation to the laboratory, coal samples were sealed with plastic bags to prevent oxidation. The collected coal sample was ground to a particle size less than 47 μm. Afterward, the mixed coal samples were thoroughly mixed and evenly divided into 4 parts. Pyrolysis experiments were conducted in a quartz tube muffle furnace and heated at a rate of 283.15 K/min to the target temperatures of 303.15 K, 523.15 K, 623.15 K, and 723.15 K. The temperature was maintained for 2 h and then naturally cooled to room temperature. The experiment was always conducted in a high-purity nitrogen (99%) atmosphere. According to the heat treatment temperature, the obtained samples were numbered. For example, R-523.15 K represents a coal sample heated to 523.15 K.

The ASTM-D5142 (moisture, volatile matter, and ash analysis), ASTM-D5373 (O, C, H, and N analysis), and ASTM-D4239 (S analysis) methods were used for approximate and ultimate analysis of heat-treated coal [41,42,43]. The average random vitrinite reflectance (VR), as an important indicator of coal metamorphism, was also determined according to GB/T 16773-2008 [44]. The studied sample (VR = 0.76%) is coal of medium rank according to ISO 11760-2005 [45].

### 2.2. XRD Test

**Figure 1:**
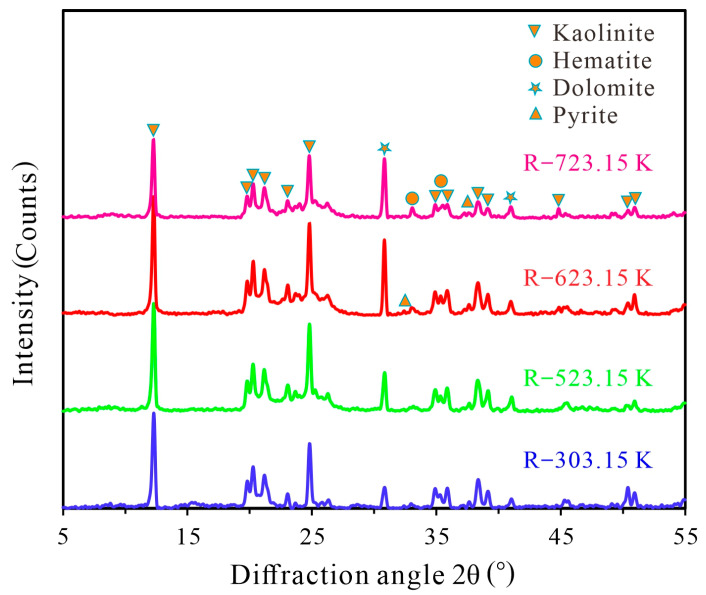
XRD diagrams of coal samples with different heat treatments.

X-ray diffractometer (D8 ADVANCE, Bruker, Germany) and Cu K_α_ radiation (40 kV, 40 mA, K_α1_ = 0.15408 nm) were used to study the mineral composition in different heat-treated coal samples (Figure 1). Within the range of 5–55°, at 5° (2θ), the sample was scanned in steps of 0.02° at a speed of min. In terms of quantitative calculation of mineral quality using self-cleaning method, JADE 6.0 software was used to comprehensively compare the main diffraction peaks (d-values and intensities) with the standard card, determine the main mineral composition based on the degree of matching, and conduct semi-quantitative calculation based on Formula (1):

(1) $$w_{0}=\left(\frac{I_{0}}{R_{0}}\right)/\left(\frac{I_{0}}{R_{0}}+\frac{I_{1}}{R_{1}}+\cdots \cdots +\frac{I_{n}}{R_{n}}\right)\times 100\%$$

*w*_0_ represents the mass fraction of the mineral phase to be tested; *I*_0_ and *R*_0_ represent the strongest peaks and RIR values of the mineral phase 0 to be measured, respectively; *I*_1_ and *R*_1_ represent the strongest peaks and RIR values of mineral phase 1 to be tested, respectively; and *I*_n_ and *R*n represent the strongest peaks and RIR values of the mineral phase n to be measured, respectively.

### 2.3. FTIR Testing and Processing

The KBr compression method was used to determine the infrared spectrum analysis of different heat-treated coal samples. To be specific, 0.005 g of the sample was mixed with 0.500 g of alkali metal halide KBr and ground in an agate mortar to 75 μm (grinding for 10 min). Then, it was put into a tablet-forming machine, pressed into pieces, and placed on the sample rack of an infrared spectroscopy testing instrument for measurement. Fourier transform infrared spectroscopy (NICOLET 5700), developed by Thermo Nicolet in the United States, was adopted to measure the spectral characteristics of the sample before and after treatment. The instrument testing conditions include a wavenumber measurement range from 400 cm^−1^ to 4000 cm^−1^, a resolution of 4 cm^−1^, a total of 32 scans, and scanning with a blank KBr film as the background to eliminate the influence of the background on spectral quality.

Due to the large particle size of different heat-treated coal samples, infrared light scattering is prone to occur, triggering a tilted baseline of the infrared spectra of different heat-treated medium-rank coals. Therefore, OPUS 8.0 software and Rubberband method should be used for baseline correction. In the original spectra of different heat-treated coal samples, the absorption bands of many functional groups in the coal contribute to the infrared spectrum, and the band is wide, which can easily lead to the phenomenon of overlapping spectral peaks. Therefore, it is difficult to determine the position of the absorption peak and the absorption intensity of functional groups within a certain region. To have a deeper understanding of the molecular structure differences of different heat-treated coals, Origin 2021 should be used to decompose the superimposed peaks and fit the original spectra. In order to achieve better fitting results, the fitting process determined the approximate position and number of initial stacked peaks based on the second-order derivatives of each spectrogram. Through selection of an appropriate peak shape function (Lorentzian or Gaussian), the least squares method was used to iteratively solve for the position, peak width, peak area, and other related parameters of each single peak [46].

### 2.4. FTIR Structural Parameters

**Figure 2:**
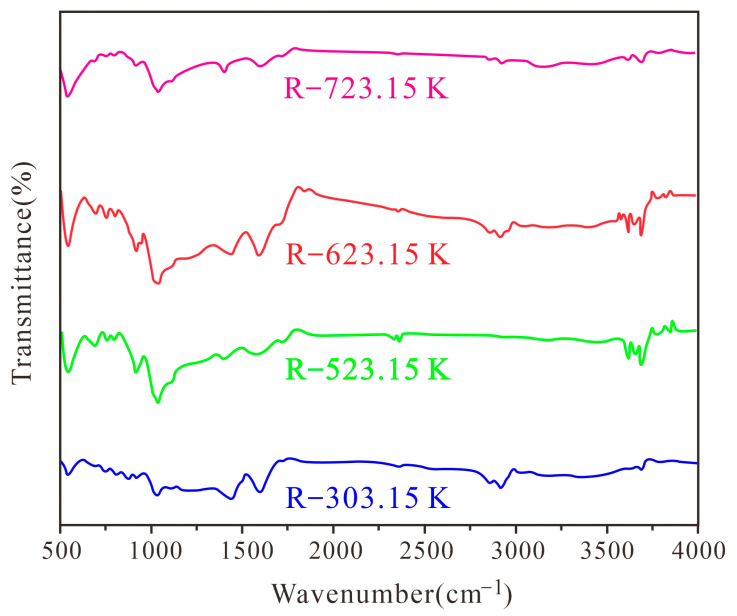
Original FTIR diagrams of coal samples with different heat treatments.

Based on FTIR structural analysis of different heat-treated coal samples (Figure 2), four infrared spectra curves were obtained. The absorption peaks of the infrared spectra of different heat-treated coal samples were distributed in four large intervals: the C-H segment of substituted aromatics ranging from 700 to 900 cm^−1^, the absorption vibration of oxygen-containing functional groups ranging from 1000 to 1800 cm^−1^, the C-H segment of fatty acids ranging from 2800 to 3000 cm^−1^, and the stretching vibration of hydroxyl and aromatic hydrocarbons ranging from 3100 to 3600 cm^−1^ [12,20,47].

Statistics on FTIR peak data of coal samples with different heat treatment was conducted. Based on the corresponding relationship between different absorption peak areas and functional group types and contents, qualitative and quantitative analysis of the molecular structure of coal samples with different heat treatments was conducted, in combination with parameters such as aromaticity, degree of condensation of aromatic rings, degree of fatty chain length and branching, maturity, aromatic hydrogen rate, and aromatic carbon rate [48]. Among them, seven structural parameters were calculated using peak area.

Aromaticity (*f*_a_), an important parameter characterizing the structural characteristic of coal molecules, represents the ratio of the number of aromatic carbon atoms in coal molecular structural units to the total number of carbon atoms [4,15]. The ratio of the relative content of the absorption band functional groups in the aromatic structure (700–900 cm^−1^) of coal molecules to the relative content of the stretching vibration band functional groups in fatty hydrocarbons (2800–3000 cm^−1^) is often used [49], as shown in Equation (2):

(2) $$f_{a}=\frac{A_{{700–900\;\mathrm{cm}}^{-1}}}{A_{2800–3000\;{\mathrm{cm}}^{-1}}}$$

Aromatic hydrogen rate (*f_H_*) represents the percentage of aromatic hydrocarbon hydrogen atoms in coal to the total number of hydrogen atoms [21,50]. It is approximately believed that only aromatic hydrogen and fatty hydrogen atoms exist in coal, as shown in Equation (3):

(3) $$f_{H}=\frac{A_{{700–900\;\mathrm{cm}}^{-1}}}{A_{2800–3000\;{\mathrm{cm}}^{-1}}+A_{{700–900\;\mathrm{cm}}^{-1}}}$$

Aromatic carbon ratio (*f_C_*) represents the percentage of carbon atoms in coal-based aromatic hydrocarbons to the total number of carbon atoms [4,15]. Myopia believes that only two carbon atoms, aromatic carbon and fatty carbon, exist in coal [51], and the calculation formula is shown in Equations (4)–(6):

(4) $$f_{C}=1-\frac{H_{\mathrm{al}}}{H}\times \frac{H}{C}\times \frac{C_{\mathrm{al}}}{H_{\mathrm{al}}}$$

(5) $$\frac{H_{\mathrm{al}}}{H}=\frac{A_{{2800–3000\;\mathrm{cm}}^{-1}}}{A_{2800–3000\;{\mathrm{cm}}^{-1}}+A_{{700–900\;\mathrm{cm}}^{-1}}}$$

(6) $$\frac{H}{C}=\frac{{12\times H}_{\mathrm{ad}}}{C_{\mathrm{ad}}}$$

*C* represents the total number of carbon atoms in the coal structure; *C_al_* represents the number of carbon atoms in fatty hydrocarbons; *H_al_*/*H* represents the ratio of fatty hydrogen to total hydrogen atoms; *H*/*C* represents the hydrogen–carbon atomic ratio, which can be calculated through elemental analysis data; and *H_al_*/*C_al_* represents the ratio of hydrogen to carbon atoms in fatty hydrocarbons, typically taken as 1.8 [46].

The degree of aromatic condensation (*D_C_*), which represents the ratio of the absorption band functional group content of the aromatic hydrocarbon structure (700–900 cm^−1^) in coal to the relative content of the vibrational functional group of the aromatic core C=C structure (1595–1605 cm^−1^), is adopted to characterize the degree of condensation between functional groups of polymer monomers [10]. The calculation is shown in Equation (7):

(7) $$D_{C}=\frac{A_{{700–900\;\mathrm{cm}}^{-1}}}{A_{1595–1605\;{\mathrm{cm}}^{-1}}}$$

The ratio of CH_2_/CH_3_ represents an important parameter that characterizes the length and branching degree of fatty chains in coal [52,53]. The ratio of the functional group content of the straight chain methylene of the fatty chain, alicyclic ring, and aromatic side chains in the wavelength range of 2900–2940 cm^−1^ to the methyl content of the branched chain of the fatty hydrocarbon side chain, alicyclic side chain, and aromatic side chain in the wavelength range of 2940~3000 cm^−1^ was used [4,54]. The calculation is shown in Equation (8):

(8) $$\frac{{\mathrm{CH}}_{2}}{{\mathrm{CH}}_{3}}=\frac{A_{{2900–2940\;\mathrm{cm}}^{-1}}}{A_{2940–3000\;{\mathrm{cm}}^{-1}}}$$

The calculation method for coal maturity (*M*) is *M* = *A*_C=C_/*A*_(C=C+COOH)_, which characterizes the changes in carboxyl C=O and C=C stretching functional groups and reflects the amount of organic matter contained in coal [10,20]. The calculation is shown in Equation (9):

(9) $$M=\frac{A_{{1595–1605\;\mathrm{cm}}^{-1}}}{A_{1595–1605\;{\mathrm{cm}}^{-1}}+A_{{1690–1715\;\mathrm{cm}}^{-1}}}$$

*IR* represents the relative content between the oxygen-containing functional group C–O and aromatic hydrocarbon C=C in coal [10], as shown in Equation (10):

(10) $$\mathrm{IR}=\frac{A_{{1120–1350\;\mathrm{cm}}^{-1}}}{A_{1550–1620\;{\mathrm{cm}}^{-1}}}$$

## 3. Results and Discussion

### 3.1. Coal Quality Analysis

According to the results of the proximate and ultimate analysis in Table 1, the moisture content ranges from 1.11% to 6.59%, and the ash yield varies from 16.87% to 24.00% in the coal samples of different heat treatments. The coal samples are highly volatile bituminous coal, with the volatile yield varying from 34.12% to 39.65%. With the increase in the processing temperature of the coal sample, the sulfur content in the coal gradually decreases while the C/H ratio gradually increases (Table 1). The side chain bond of sulfur-containing fat in coal is broken, and pyrite is decomposed during the heating process of coal, reducing the sulfur content in coal [55,56,57].

### 3.2. Mineralogical Characterization

**Figure 3:**
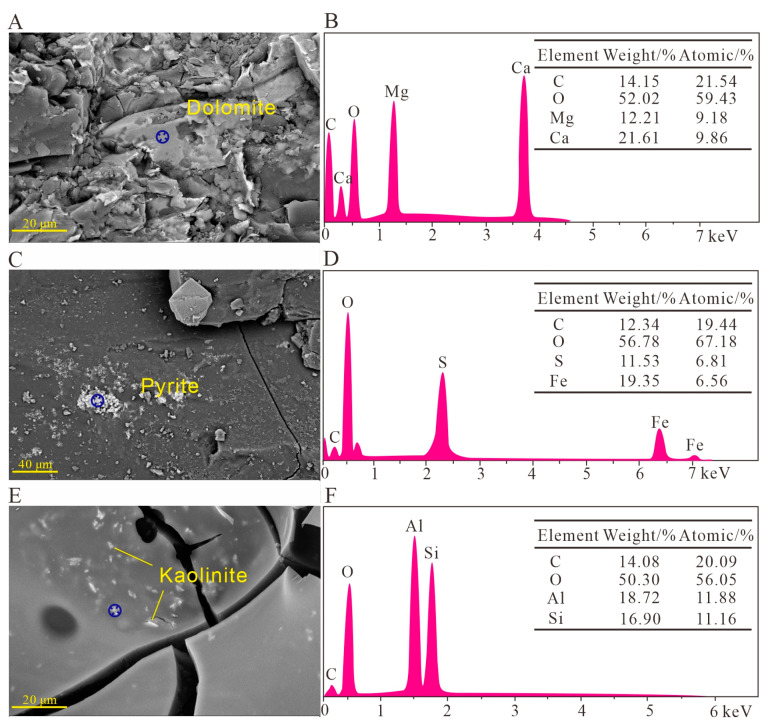
Mineral composition in coal samples with different heat treatments under scanning electron microscopy and energy dispersive spectroscopy. (**A**,**B**) R-523.15 K sample; (**C**,**D**) R-303.15 K sample; (**E**,**F**) R-723.15 K sample.

An X-ray diffractometer was used to analyze the mineral composition of coal samples under different heat treatments (see the results in Table 2). In the unheated coal sample, the mineral phases are mainly dolomite (Figure 3A,B), pyrite (Figure 3C,D), and kaolinite (Figure 3E,F). With the increase in the heat treatment temperature, the peak intensity of kaolinite and pyrite gradually decreases. In addition, the peak intensity of dolomite and hematite gradually increases (Figure 2). Therefore, during the heating process, the mineral phases in coal samples, such as dolomite and hematite, which are filled in cell cavities or pores, are released and processed. Kaolinite undergoes a transformation into metakaolinite during the heat treatment [58] until the processing temperature reaches 1173.15 K, where all diffraction peaks of kaolinite disappear, and new minerals such as hematite are produced [59]. In the heating process, pyrite produces hematite and magnetite. However, no magnetite is found in the test results [58]. The details of the conversion reaction between pyrite and kaolinite under heating are shown in Formulas (11) and (12).

Pyrite (FeS_2_) + O_2_ → Hematite (Fe_2_O_3_) + Magnetite (Fe_3_O_4_) + SO_2_↑ (523.15–1173.15 K) (11)

Clay minerals → Dehydration + Dehydroxylation (T < 873.15 K)(12)

### 3.3. FTIR Spectroscopy Analysis

#### 3.3.1. Peak Fitting and Analysis of Hydroxyl Structure

**Figure 4:**
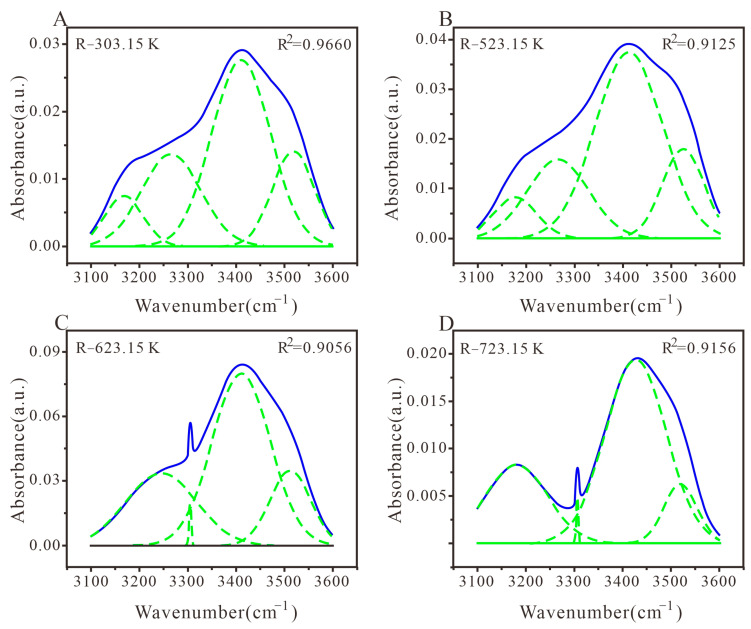
FTIR plots of hydroxyl structure peak fitting in coal samples with different heat treatments. (**A**) Coal samples treated at 303.15 K; (**B**) Coal samples treated at 523.15 K; (**C**) Coal samples treated at 623.15 K; (**D**) Coal samples treated at 723.15 K.

The hydroxyl group is not only one of the active functional groups in characterizing the coal oxidation process but also a crucial functional group in analyzing the molecular structure of coal [60]. Four sub-peaks were used to fit the hydroxyl structure (3100–3600 cm^−1^), with a fitting coefficient of R^2^ ranging from 90.56% to 96.60%. The overall fit degree of the four different heat-treated coals is lower than that of aromatic and fatty hydrocarbon structures, which is mainly attributed to the impact of more vibrational peaks on the hydroxyl group in the FTIR diagram of the experimental sample. As shown in Figure 4A,B, the R-303.15 K and R-523.15 K samples exhibit a large and wide absorption peak near 3410 cm^−1^, representing self-associating hydroxyl groups. As revealed in Figure 4C,D, there is a small and narrow absorption peak near 3305 cm^−1^, which represents ether oxyhydroxyl groups [19].

As shown in Figure 4 and Table 3, the hydroxyl structure of medium-rank coal with different heat treatments mainly consists of self-associating hydroxyl groups, and the degree of condensation of functional groups in coal char is strong, mainly composed of hydroxyl polymers. With the increase in heat treatment temperature, the self-associated hydroxyl groups in medium-rank coal gradually increase, while the ether oxygen hydroxyl groups gradually decrease, indicating that higher temperatures can increase the degree of aromatic ring condensation in medium-rank coal and thus accelerate the formation of self-associated hydrogen bonds. The contribution of hydrogen bonds in the 3600–3100 cm^−1^ region of the infrared spectrum decreases, revealing a decrease in the number of hydroxyl groups [61].

#### 3.3.2. Peak Fitting and Analysis of C-Hal Structure

**Figure 5:**
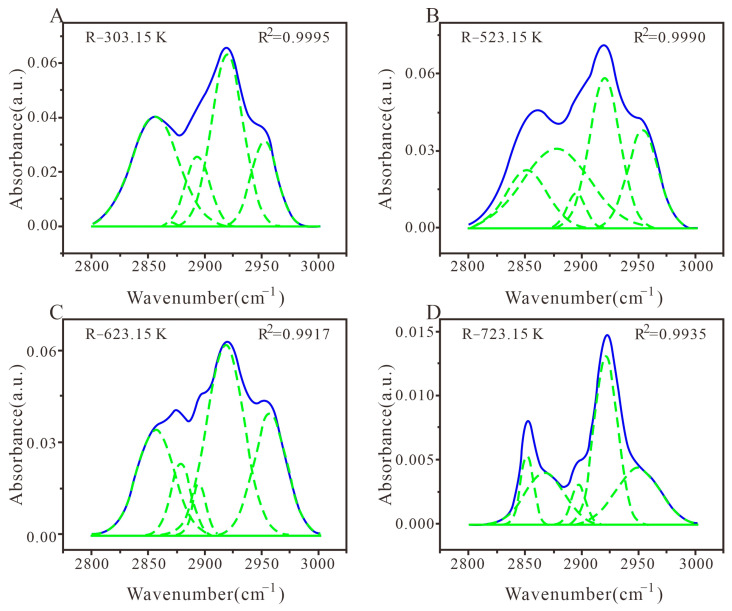
FTIR plots of fatty hydrocarbon structure peak fitting in coal samples with different heat treatments. (**A**) Coal samples treated at 303.15 K; (**B**) Coal samples treated at 523.15 K; (**C**) Coal samples treated at 623.15 K; (**D**) Coal samples treated at 723.15 K.

There are three types of fatty substances in different heat-treated coals: –CH_3_, –CH_2_–, and C–H, with wave numbers ranging from 2800 cm^−1^ to 3000 cm^−1^. The spectra of different heat-treated medium-rank coal samples in this band were fitted using the five sub-peaks [62], and the determination coefficient R^2^ ranges from 99.17% to 99.95%. As shown in Figure 5A,B, strong absorption peaks appear near 2850 cm^−1^ and 2920 cm^−1^, representing symmetric –CH_2_– stretching vibration and asymmetric –CH_2_– stretching vibration, respectively. Strong absorption peaks appear near 2895 cm^−1^ and 2950 cm^−1^, representing methane C-H stretching vibration and asymmetric -CH_3_ stretching vibration, respectively [39]. The vicinity of 2870 cm^−1^ represents the symmetric –CH_3_ stretching vibration (Figure 5C,D). Overall, R-623.15 K exhibits stronger asymmetric –CH_2_– and –CH_3_ stretching vibration in comparison to other medium-rank coals.

As shown in Figure 5 and Table 4, in the structure of fatty hydrocarbons, R-303.15K has the highest relative content of –CH_2_–, while the relative content of –CH_3_ and C–H is relatively low. With the increase in heat treatment temperature, the relative content of asymmetric –CH_3_ vibration gradually increases, and the relative content of –CH_2_– and –CH_3_ presents irregular changes [52,62]. This is because the temperature causes the continuous fracture of long chains in fatty hydrocarbons, inducing a gradual reduction in long chains and an increase in branched chains [4,63].

#### 3.3.3. Peak Fitting and Analysis of Oxygen Functional Groups

**Figure 6:**
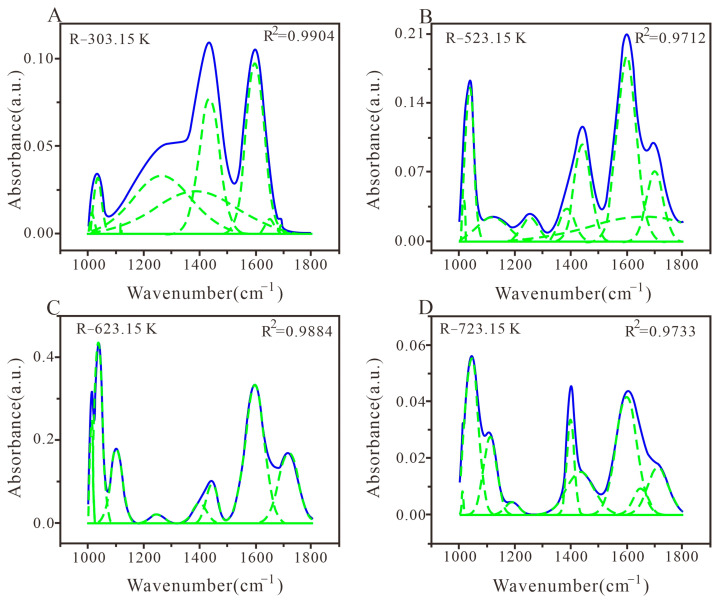
FTIR plots of peak fitting of oxygen-containing functional group structures in coal samples with different heat treatments. (**A**) Coal samples treated at 303.15 K; (**B**) Coal samples treated at 523.15 K; (**C**) Coal samples treated at 623.15 K; (**D**) Coal samples treated at 723.15 K.

In this study, nine sub-peaks were used to fit the absorption vibration of oxygen-containing functional groups (1000–1800 cm^−1^) in different heat-treated coals [64], with a fitting coefficient R^2^ ranging from 97.12% to 99.04%. As shown in Figure 6A, due to the cross-influence and overlap between functional groups, there are three peaks in the 1030–1350 cm^−1^ range, namely 1034 cm^−1^, 1117 cm^−1^, and 1265 cm^−1^, belonging to the C–O stretching vibration of oxygen-containing functional groups such as alcohols, phenols, ethers, phenoxy groups, acids, and lipids. Apart from that, there is a symmetric deformation –CH_3_ vibration at 1385 cm^−1^ and an aliphatic chain (–CH_3_, –CH_2_–) asymmetric vibration at 1435 cm^−1^. In Figure 6B, the ash content is present at 1012 cm^−1^, the conjugated C=O vibration is present at 1659 cm^−1^, and the stretching vibration of carboxyl C=O is present at 1699 cm^−1^. Obviously, there is alkyl ether at 1035 cm^−1^ and aryl ether stretching vibration at 1101 cm^−1^ (Figure 6C). As shown in Figure 6D, alkyl ether is present at 1043 cm^−1^, and the C=C stretching vibration of aromatic hydrocarbons is present at 1598 cm^−1^. Through comparison, it could be found that with the increase in the temperature, the oxygen content in coal char first increases and then decreases, and the absorption frequency gradually moves towards the low wavenumber range [65].

There are many peak positions in the range of 1000–1800 cm^−1^, with the majority of oxygen-containing functional groups, but there is also a small amount of ash (Figure 6 and Table 5). In addition, R-303.15 K coal has many peak positions in the range of 1000–1800 cm^−1^, including phenol, alcohol, ether, phenolic deformation C–O (stretching), symmetric deformation –CH_3_ (bending), aliphatic chains (–CH_3_, –CH_2_–), and aromatic nucleus C=C vibration, with a relatively high content of conjugated C=O, aliphatic chains (–CH_3_, –CH_2_–), and aromatic carboxyl C=O. In other coal chars, the relative contents of aryl ether, alkyl ether, aromatic nucleus C=C vibration, and aromatic carboxyl C=O are higher. With the increase in heat treatment temperature, the content of the aromatic carboxyl C=O and aromatic nucleus C=C in coal first increases and then decreases. The reason is that the dehydrogenation of cycloalkanes during the heating process of coal enhances the degree of coal aromatization, triggering an increase in the aromatic nucleus C=C. However, during the metamorphism process, the accumulation of heat due to oxidation and heating enhances the pyrolysis effect, inducing a decrease in the aromatic nucleus C=C. This also reduces the stretching vibration content of phenols, alcohols, ethers, and esters C–O [66].

#### 3.3.4. Peak Fitting and Analysis of Aromatic Structure

**Figure 7:**
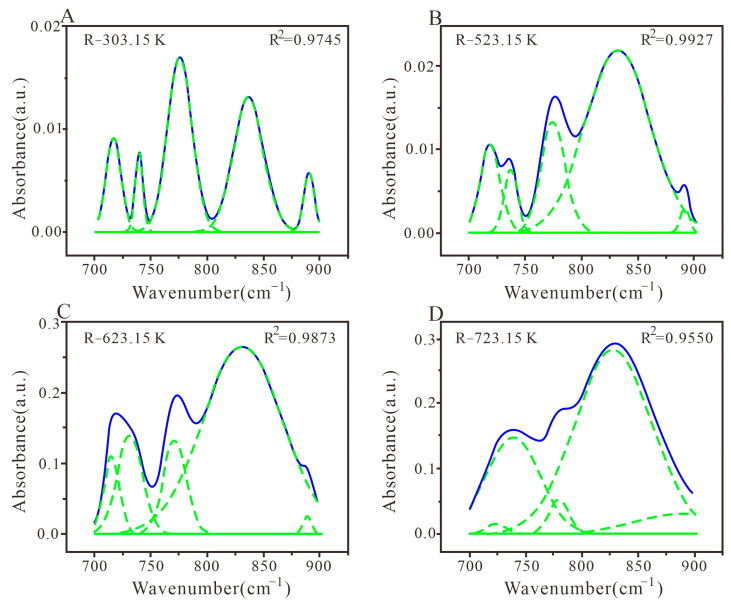
FTIR plots of aromatic hydrocarbon structure peak fitting in coal samples with different heat treatments. (**A**) Coal samples treated at 303.15 K; (**B**) Coal samples treated at 523.15 K; (**C**) Coal samples treated at 623.15 K; (**D**) Coal samples treated at 723.15 K.

The wave number range of 700–900 cm^−1^ in coal characterizes the absorption vibration of aromatic hydrocarbon structures. Within this range, different heat-treated coal chars exhibit different results, as shown in Figure 7. In order to achieve better fitting results, the five sub-peaks are used for fitting, and the coefficient of determination R^2^ reaches over 95%. The position of the absorption peak is related to the number of adjacent H on the benzene ring, and the type of substitution of aromatic rings in the general coal molecular structure can be inferred from the distribution of adjacent H [67]. In general, there are four substitution methods for coal aromatic hydrocarbon structures: disubstituted benzene ring in the wave number range of 730–750 cm^−1^, trisubstituted benzene ring in the wave number range of 750–810 cm^−1^, four substituted benzene rings in the wave number range of 810–850 cm^−1^, and pentasubstituted benzene ring in the wave number range of 850–900 cm^−1^ [67,68]. Based on the peak area in the wave number range of 700–900 cm^−1^ for the absorption vibration of aromatic hydrocarbon structures, two obvious absorption peaks are located near 774 cm^−1^ and 837 cm^−1^, representing three adjacent H deformations and two adjacent H deformations, accounting for approximately 39.22% and 37.21%, respectively (Figure 7A). As shown in Figure 7B, there is a wide and large absorption peak near 832 cm^−1^, accounting for approximately 68.42%, which is two adjacent H deformations (aromatic nucleus, C–H). As shown in Figure 7C, two obvious absorption peaks are between 731 cm^−1^ and 771 cm^−1^, representing four adjacent H deformations and three adjacent H deformations, respectively. As revealed in Figure 7D, there is a large and wide absorption peak near 827 cm^−1^, as well as a more obvious absorption peak at 739 cm^−1^, accounting for approximately 66.71% and 24.44%, respectively. As the temperature increases with different heat treatments, the structural components of aromatic hydrocarbons first increase and then decrease, while the number of trisubstituted benzene ring compounds gradually decreases, indicating that the absorption intensity changes with the increase in the temperature [69].

As shown in Table 6 and Figure 7, the aromatic hydrocarbon structure in R-303.15 K coal mainly consists of a trisubstituted benzene ring and four substituted benzene rings. However, in other coal samples, the four substituted benzene rings are dominant, and the pentasubstituted benzene ring is less. With the increase in heat treatment temperature, the disubstituted benzene ring presents an upward trend (except for R-303.15 K), while the trisubstituted benzene ring presents a downward trend. The structural changes in aromatic hydrocarbons in coal are mainly attributed to the deoxygenation and aromatization of cycloalkanes, which change the degree of condensation of aromatic hydrocarbons [48,52,66].

#### 3.3.5. Variation of Molecular Structure Parameters of Coal

The *f_a_*, *f*_H_*,* and *f_c_* present a trend of first increasing and then decreasing with the temperature increase in different heat treatments (Table 7). The reason is that during the heating process of coal, cycloalkanes will dehydrogenate and aromatize and reach a high degree of aromatization at 623.15 K. This further indicates that temperature is crucial for the aromatization of coal. Subsequently, With the continuous increase in temperature, the activation energy of the coal sample combustion reaction decreases, leading to a decrease in aromaticity [48,70].

The CH_2_/CH_3_ value gradually decreases with the temperature increase in different heat treatments (Table 7), indicating that the length of the fat side chain becomes shorter and the number of branch chains increases [66]. As for the heating process of coal, it exhibits the process of continuously breaking the fatty chain, decreasing the number of methylene groups, and increasing the number of methyl groups to form more short-branched chains [12,71].

With the increase in temperature of the heat-treated coal sample, the *IR* presents a trend of first decreasing and then increasing (Table 7). This is because the coal removes the C–O oxygen-containing functional groups, and the aromatics are destroyed in the later stage during the heating process [66]. The maturity of organic matter (*M*) presents a trend of first decreasing and then increasing with the temperature increase in heat-treated coal (Table 7), indicating that the maturity of organic matter in coal gradually matures. However, due to the continuous increase in carbon content in coal, the variation in organic matter requires higher thermal energy [39,72]. Meanwhile, the degree of condensation (*D_C_*) of the coal samples with different heat treatments is much higher than that of the raw coal (R-303.15 K) (Table 7) because unstable carboxyl and carbonyl oxygen-containing functional groups are continuously removed during the heating process [15,73].

### 3.4. Implications for Gasification

The conventional gases in coal pyrolysis mainly include CH_4_, C_2_H_2_, H_2_O, H_2_, CO_2_, and CO. However, there are some differences in the content of total sulfur and form sulfur in the raw coal, triggering significant variations in the escape of different sulfur elements during the pyrolysis process of the coal sample [74]. In order to reasonably utilize high-sulfur coal resources, reduce the sulfur content in coal, and meet the production needs of blast furnaces, the sulfur conversion process should be controlled through coal washing and specific pyrolysis process conditions [75].

Based on FTIR technology, the molecular structure of coal samples after different heat treatments was studied, and the quality of coal was preliminarily evaluated, indicating the differences in basic properties and elemental composition of coal. Through the approximate analysis and limit analysis of some empirical equations, some parameters representing molecular structural characteristics, such as *f*_H_, *f*_C_, *f*_a_, *IR*, and *D*c, were calculated. These parameters indicate that with the increase in heat treatment temperature, organic components in coal are gasified and precipitated, such as dehydrogenation and aromatization of cycloalkanes in coal, shortening of fatty chains, and reduction in oxygen-containing functional group content.

Overall, FTIR achieved certain results in studying the molecular structure of coal samples under different heat treatments. It is noteworthy that due to the inherent complexity of coal gasification products with different coal qualities and metamorphic degrees, detailed, accurate, and precise analysis of macromolecular structures is quite challenging. Therefore, in the analysis of highly heterogeneous coal gasification samples, it is recommended to combine various techniques such as 13C NMR, XRD, and TG-MS to acquire semi-quantitative structural information of molecules in coal samples from different perspectives.

## 4. Conclusions

(1) With the increase in the heat treatment temperature, the sulfur content in coal gradually decreases, the C/H ratio gradually increases, and the content of kaolinite and pyrite in coal gradually decreases, but the content of dolomite and hematite gradually increases.

(2) There are significant differences in the molecular structures of different heat-treated coals. The relative content of ether oxygen hydroxyl groups in medium-rank coal with high heat treatment temperature significantly decreases, while the relative content of self-associated hydroxyl groups and cyclic hydroxyl groups increases. In addition, the relative content of methylene in the fatty hydrocarbon structure is relatively high, and the intensity of the absorption peak of asymmetric stretching vibration of methyl groups is enhanced.

(3) With the increase in heat-treated temperature, the infrared spectral area of oxygen-containing functional groups presents a trend of first increasing and then decreasing. Among them, the peak area of aromatic nucleus C=C vibration is the largest (except for R-303.15 K), and its relative content presents a trend of first increasing and then decreasing, while the oxygen-containing functional groups of heat-treated coal samples basically do not contain phenolic deformation C–O (stretching). With the increase in heat treatment temperature, the relative concentrations of alkyl ether (C–O) increase. The structure of aromatic hydrocarbons mainly consists of four substituted benzene rings (except for R-303.15 K). With the increase in the temperature, the relative content of the trisubstituted benzene ring decreases.

(4) With the increase in heat treatment temperature, *f_a_*, *f*_H_*, f_c_*, and *Dc* in medium-rank coals present a trend of first increasing and then decreasing, *M* and *IR* present a trend of first decreasing and then increasing, and CH_2_/CH_3_ gradually decreases. This further indicates that the length of the fatty side chains in the fatty hydrocarbon structure of medium-rank coals becomes shorter, the number of branch chains increases continuously, and the aromaticity and condensation degree are significantly improved. On this basis, further research on the effect of coal gasification suggests combining various technologies such as 13C NMR, XRD, and TG-MS to obtain semi-quantitative structural information of molecules in coal from different perspectives.

## Figures and Tables

**Table 1:** The ultimate and proximate analysis results of coal samples.

Numbers	Moisture	Ash	Volatile Matter	C	H	O	N	S	C/H
R-303.15 K	1.11	16.87	39.65	80.96	5.28	8.42	1.59	3.11	15.33
R-523.15 K	4.19	17.70	34.12	72.30	3.09	19.81	1.57	2.65	23.40
R-623.15 K	6.59	27.41	38.03	69.13	2.37	23.73	2.45	1.68	29.17
R-723.15 K	6.06	24.00	39.25	70.12	1.99	23.18	2.52	1.66	35.24

**Table 2:** The mineralogical analysis results of coal samples (%).

Numbers	Kaolinite	Dolomite	Hematite	Pyrite
R-303.15 K	86.2	11.7	0.2	1.9
R-523.15 K	75.2	22.7	1.2	0.9
R-623.15 K	70.4	26.6	2.4	0.6
R-723.15 K	65.7	27.4	6.5	0.4

**Table 3:** Results of hydroxyl structure in different heat-treated medium-rank coals.

Functional Groups	R-303.15 K			R-523.15 K			R-623.15 K			R-723.15 K		
	PP	PA	P	PP	PA	P	PP	PA	P	PP	PA	P
AHG	3518	1.49	17.57	3525	1.95	15.87	3512	3.66	16.56	3519	0.54	11.05
SHG	3411	4.20	49.45	3413	2.52	55.34	3411	12.07	55.63	3426	3.07	63.69
EHG	3266	2.09	24.62	3266	6.54	21.30	3305	0.10	0.48	3306	0.03	0.55
CHG	3168	0.71	8.36	3178	0.92	7.49	3246	6.05	27.33	3180	1.32	24.71

AHG, aromatic hydroxyl group; SHG, self-associating hydroxyl group; EHG, etheroxy hydroxyl group; CHG, cyclic hydroxyl group; PP, peak position (cm^−1^); PA, peak area; and P, percentage (%).

**Table 4:** Results of fatty hydrocarbons in medium-rank coal samples with different heat treatments.

Functional Groups	R-303.15 K			R-523.15 K			R-623.15 K			R-723.15 K		
	PP	PA	P	PP	PA	P	PP	PA	P	PP	PA	P
S-CH_2_	2856	2.13	35.60	2851	1.02	14.89	2856	1.41	23.13	2851	0.08	8.88
S-CH_3_	2870	0.02	0.27	2877	2.26	33.08	2879	0.53	8.69	2866	0.18	20.80
M-CH	2893	0.70	11.71	2894	0.28	4.05	2895	0.24	3.95	2896	0.05	5.73
A-CH_2_	2919	2.21	37.06	2920	1.98	28.95	2918	2.49	40.77	2921	0.34	39.43
A-CH_3_	2952	0.92	15.36	2953	1.30	19.03	2957	1.43	23.46	2949	0.22	25.15

S-CH_2_, symmetric –CH_2_– stretching vibrations; S-CH_3_, symmetric –CH_3_ stretching vibration; M-CH, methane C–H stretching vibration; A-CH_2_, asymmetric –CH_2_– stretching vibrations; and A-CH_3_, asymmetric –CH_3_ stretching vibration.

**Table 5:** Results of oxygen-containing functional groups in medium-rank coal samples with different heat treatments.

Functional Groups	R-303.15 K			R-523.15 K			R-623.15 K			R-723.15 K		
	PP	PA	P	PP	PA	P	PP	PA	P	PP	PA	P
Ash	1015	0.19	0.54	1012	0.52	1.00	1010	2.71	3.32	1012	0.07	0.45
Alkyl ether (C–O)	1034	1.07	3.10	1034	6.06	11.43	1035	14.77	18.33	1043	3.24	22.59
Aryl ether (C–O)	1117	0.00	0.00	1117	3.36	6.34	1101	9.22	11.51	1111	1.64	11.44
Phenolic deformation C–O (stretching)	1265	8.77	25.48	1253	1.82	3.50	1247	1.22	1.52	1189	0.28	1.93
Symmetric deformation –CH_3_ (bending)	1385	8.93	25.92	1385	2.22	4.26	1399	2.94	3.66	1399	1.10	7.65
Aliphatic chains (–CH_3_, –CH_2_–)	1435	7.24	21.16	1441	7.21	13.84	1443	4.64	5.79	1433	1.88	13.08
Aromatic nucleus (C=C)	1599	7.84	22.89	1599	16.32	31.32	1597	29.12	36.34	1598	3.95	27.58
Conjugate (–C=O)	1651	0.29	0.84	1659	12.35	18.20	1662	1.08	1.35	1650	0.63	4.40
Aromatic carboxyl (C=O)	1693	0.03	0.08	1699	5.27	10.11	1717	14.68	18.17	1712	1.56	10.87

**Table 6:** Results of aromatic hydrocarbons in coal samples with different heat treatments.

Functional Groups	R-303.15 K			R-523.15 K			R-623.15 K			R-723.15 K		
	PP	PA	P	PP	PA	P	PP	PA	P	PP	PA	P
DBR	714	0.15	12.69	718	0.91	9.94	714	1.87	5.82	723	0.03	0.93
	737	0.07	12.60	737	0.43	4.76	731	3.91	10.37	739	0.86	23.50
TBR	774	0.48	39.22	774	1.42	15.61	771	3.33	10.36	779	0.11	3.26
FSBR	837	0.45	37.21	832	6.28	68.42	831	23.36	71.00	827	2.33	66.71
PBR	891	0.07	5.51	891	0.12	1.26	889	0.22	0.68	889	0.20	5.59

DBR, disubstituted benzene ring; TBR, trisubstituted benzene ring; FSBR, four substituted benzene rings; and PBR, pentasubstituted benzene ring.

**Table 7:** Molecular structure parameters of coal samples with different heat treatments.

Parameters	R-303.15 K	R-523.15 K	R-623.15 K	R-723.15 K
*f*_H_	0.235	0.573	0.843	0.805
*f*_C_	0.667	0.878	0.964	0.963
*f_a_*	0.307	1.340	5.357	4.126
CH_2_/CH_3_	2.411	1.521	1.736	1.562
*IR*	1.120	0.112	0.042	0.071
*M*	0.996	0.756	0.665	0.717
*D_C_*	0.234	0.561	1.122	0.896

## Data Availability

The data supporting the findings of this study are available within the article.
